# Financial Impact of Complex Cancer Surgery in India: A Study of Pancreatic Cancer

**DOI:** 10.1200/JGO.17.00151

**Published:** 2018-05-03

**Authors:** Guruchanna Basavaiah, Priyanka D. Rent, Eugene G. Rent, Richard Sullivan, Margaret Towne, Marieke Bak, Bhawna Sirohi, Mahesh Goel, Shailesh V. Shrikhande

**Affiliations:** **Guruchanna Basavaiah**, **Mahesh Goel**, and **Shailesh V. Shrikhande**, Tata Memorial Centre, Mumbai; **Priyanka D. Rent**, K.S. Hegde Medical Academy, Mangalore; **Eugene G. Rent**, A.J. Hospital and Research Centre, Mangalore, India; **Richard Sullivan**, King’s College London, Guys and St Thomas’ NHS Foundation Trust; **Margaret Towne**, London School of Hygiene & Tropical Medicine; **Bhawna Sirohi**, Barts Cancer Institute, London, United Kingdom; and **Marieke Bak**, VU University, Amsterdam, Netherlands.

## Abstract

**Purpose:**

The rapidly increasing burden of cancer in India has profound impacts on health care costs for patients and their families. High out-of-pocket (OOP) expenditure, lack of insurance, and low government expenditure create a vicious cycle, leading to household impoverishment. Complex cancer surgery is now increasingly important for emerging countries; however, little is understood about the macro- and microeconomics of these procedures. After the Lancet Oncology Commission on Global Cancer Surgery, we evaluated the OOP expenditure for patients undergoing pancreatico-duodenectomy (PD) at a government tertiary cancer center in India.

**Methods:**

Prospective data from 98 patients who underwent PD between January 2014 and June 2015 were collected and analyzed. The time frame for consideration of expenses, including all preoperative investigations, was from the first hospital visit to the day of discharge. Catastrophic expenditure was calculated by assessing the percentage of households in which OOP health payments exceeded 10% of the total household income.

**Results:**

The mean expenditure for PD by patients was Rs.295,679.57 (US$74,420, purchasing power parity corrected). This amount was significantly higher among those admitted to a private ward and those with complications. Only 29.6% of the patients had insurance coverage. A total of 76.5% of the sample incurred catastrophic expenditure, and 38% of those with insurance underwent financial catastrophe compared with 93% of those without insurance. The percentage of patients facing catastrophic impact was highest among those in semiprivate wards, at 86.7%, followed by those in public and private wards.

**Conclusion:**

The cost of PD is high and is often unaffordable for a majority of India’s population. A review of insurance coverage policies for better coverage must be considered.

## INTRODUCTION

The incidence of cancer is on the rise globally. In India alone, 700,000 deaths are attributed to cancer every year.^1,2^ This rise in cancer incidence translates into increased health care costs in terms of treatment, lost earnings, and the rehabilitation of patients, which can be protracted in cancer care. This problem of high health care expenditure is more pronounced in emerging powers such as India, where insurance coverage is not comprehensive and government total health expenditure (1.4% of GDP in 2014)^3^ is limited. Only 25% of India’s population is covered under any sort of health insurance, which includes all forms of private, employer-based, and government-sponsored health insurance schemes.^4^ As a result, a cancer diagnosis has a huge impact on personal finances, and with increasing needs for complex cancer surgery, the cost impact is set to rise, necessitating a greater understanding of the total economic impact in national settings.^5,6^

Surgeries for pancreatic cancer, one of the bellwether cancers for complex tertiary care, are major procedures, with their own set of complications. Increased morbidity is associated with increased costs of treatment because the costs incurred to treat complications further add to personal and institutional costs.^7^ A recent series from India showed that despite excellent results and a low mortality rate, one third of patients undergoing pancreatico-duodenectomy (PD) have significant morbidity.^8^

From a public health as well as a policy perspective, it is imperative to have reliable data on the economic burden of cancer, particularly on complex and expensive surgical procedures undertaken in the public sector, so that evidence-based decisions regarding allocation of resources to public health programs can be made. Hence, the aim of this study was to provide data regarding the economic burden faced by patients undergoing PD, to aid in the making of policy decisions and in planning, as well as to provide benchmarks for the prioritization of resources and sufficient protection against catastrophic expenditure.

## METHODS

Data were obtained prospectively between January 2014 and June 2015 to assess the costs incurred for PD at Tata Memorial Centre, India’s major tertiary cancer center. The center is a public entity and receives partial support from the Government of India for research and treatment. The study was approved by the institutional review board of Tata Memorial Centre after the board reviewed the study protocol and the feasibility of the study. A questionnaire was developed on the basis of a review of previous studies that attempted to measure the expenditure incurred by patients while accessing treatment, covering all the categories under which patients were expected to incur expenses for surgical care. The questionnaire was analyzed by experts familiar with the subject and by our institutional review board, which reviewed all the questionnaire items for comprehensiveness and readability. The questionnaire was edited on the basis of their recommendations. This study was objective in nature (ie, patients stated the amount they paid for a particular service); in this type of study answers may vary greatly, and therefore, we expected high variability in the data. 

The Comprehensive Score for Financial Toxicity tool for measuring the financial distress experienced by patients with cancer has been validated in a study conducted in the United States. Although validated and largely applicable to the Indian population, the study was published in June 2014, whereas data collection for this study commenced in January 2014; hence, we were unable to use this tool for the study.^9^ The questionnaire was distributed to patients who underwent a PD, and was to be completed either by the patient him- or herself or by any family member who was knowledgeable about hospitalization expenses. The time frame during which expenses were considered was from the first hospital visit to postoperative recovery (ie, until the day of discharge) and included charges of preoperative investigations. The questionnaire contained questions on expenditure during each stage of disease management, including baseline and preoperative blood tests, radiologic imaging, surgical costs including consumables, surgery charges, and intensive care unit and room charges until the day of discharge. The cost of chemotherapy and radiotherapy and any type of indirect cost were not included in the study. Informed consent was obtained from all patients after they had reviewed the patient information leaflet. Patients were asked to post back the questionnaires after discharge or to hand them over to the physician during their first follow-up visit. For patients who failed to return the questionnaire, telephonic interviews were conducted with either the patient or a first-degree relative.

To assess the extent of financial burden on the households in our study, we calculated the incidence of catastrophic expenditure among the households in which a member had undergone PD. Catastrophic expenditure was calculated by assessing the percentage of households whose out-of-pocket (OOP) health payments (Hexp) exceeded a certain percentage (Zcat) of the household’s total prepayment annual income (x), used as a proxy for the household’s capacity to pay. Therefore, OOP expenditure is considered catastrophic when Hexp/x exceeds a specified threshold (ie, Zcat).^10,11^ When households’ total annual income is used as a denominator for calculating Zcat, the most common threshold set is 10%; hence, the catastrophic threshold for our study was set at 10%.^10,12-14^ The underlying ideology in setting the threshold for defining catastrophic expenditure is that if a household’s expenditure on health care is more than the threshold, the household will have to reduce its expenditure on other subsistence needs because of the need to pay for health care. This may push households into poverty or deepen their existing poverty.^10,11,15^ Currency conversion was at the rate of $US1 = 68 Indian Rupee (INR; Rs) as of January 1, 2015. The data on expenses have been mentioned in both the crude US dollar value and the purchasing power parity (PPP)–corrected value for ease of understanding.^16^ PPP conversions were performed according to standard protocols for January 1, 2015.^17^ PPP reflects a country’s ability to purchase a standardized set of goods and services, facilitating comparisons across countries. Thus, the PPP standardizes costs across countries using a common reference point: the US dollar. The US dollar, as the reference currency, is equal to unity. Reporting cost information in a common currency is a standard approach in health economics and one that is recommended by WHO.

## RESULTS

The analysis was based on data collected from 98 patients (mean age, 54.5 years [range, 10 to 87 years]) who underwent PD; 66.3% of the respondents were male; 45.9% were public, 30.6% semiprivate, and 23.5% private ward admissions. Table 1 documents the demographic details of the patients.

**Table 1:**
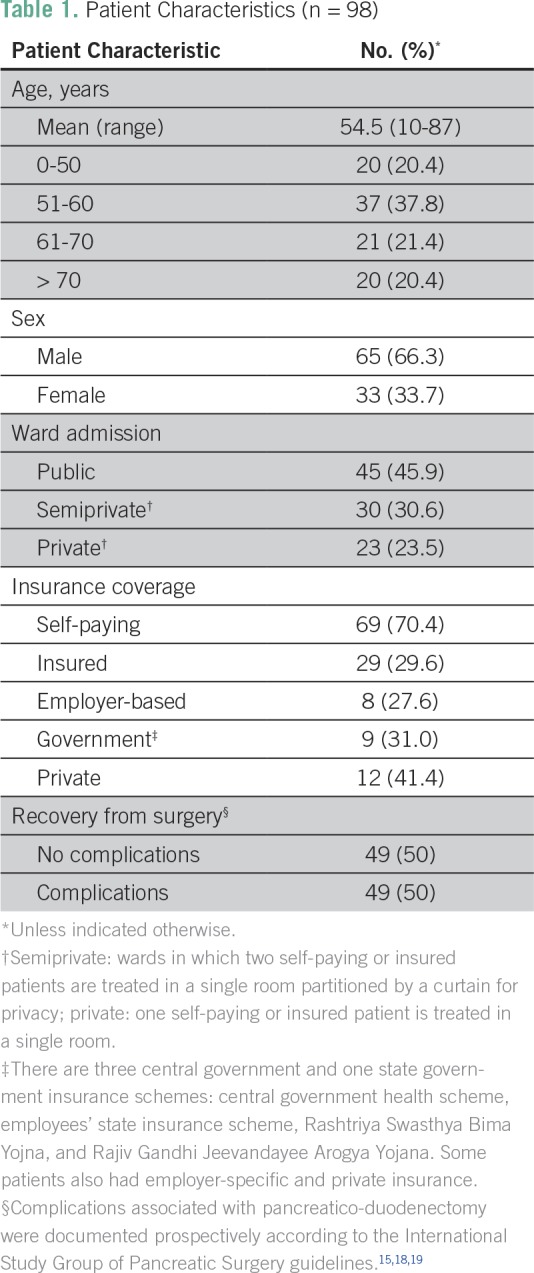
Patient Characteristics (n = 98)

Among the 98 patients, only 29.6% had insurance coverage, whereas the rest had to make OOP payments for hospitalization. It was observed that, if segregated according to the type of ward to which the patient was admitted, 40% of those admitted to a semiprivate ward had insurance coverage compared with 30.4% of those admitted to private and 22.2% to public wards. Among those who were insured, 41.4% had private insurance, 31% had government insurance, and 27.6% had employer-based insurance.

Table 2 lists the types of insurance coverage among the insured patients, segregated by the ward to which they were admitted. Among patients in public wards, 90% were covered by a government-based insurance scheme.

**Table 2:**
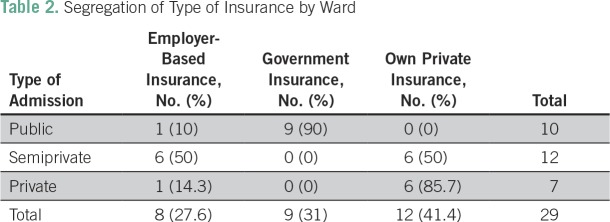
Segregation of Type of Insurance by Ward

The mean household income of patients in semiprivate wards was slightly higher than that of those in private wards; however, on assessing the median, we see that those admitted in the public ward had the lowest family income.

The expenditure incurred by patients differed according to different parameters such as type of ward, insurance coverage, presence of complications, and sex. Table 3 lists the mean and median expenditure incurred by patients in each subcategory.

**Table 3:**
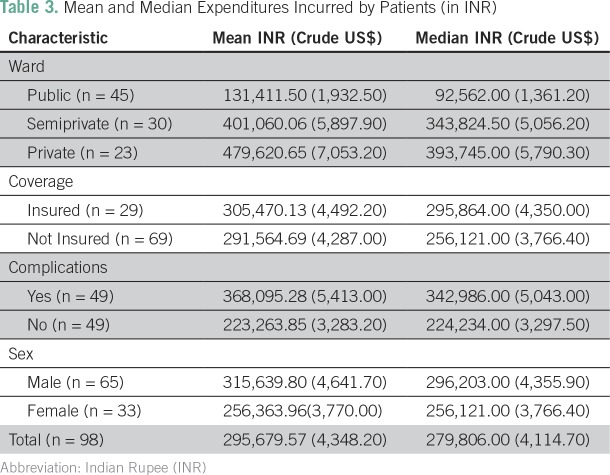
Mean and Median Expenditures Incurred by Patients (in INR)

The mean expenditure of patients admitted to private and semiprivate wards was significantly higher (*P* < .001) than the mean expenditure of those admitted to public wards. Although the mean expenditure of those with insurance was slightly higher than those without insurance, this difference was not statistically significant.

The mean expenditure for patients with complications was significantly higher (*P* < .005), at Rs.368,095.28 (US$5,413 crude; US$92,649 PPP corrected) than that of those without complications, which was Rs.223,263.85 (US$3,283 crude; US$56,192 PPP corrected).^18-20^ The mean expenditure for male patients was found to be higher, at Rs.315,639.8 (US$4,642 crude; US$79,452 PPP corrected) than that of female patients, although no statistical significance could be established.

As seen in Table 4, if a comparison is made between the mean expenditure for patients with and without insurance admitted to the different wards, we see that the mean expenditure for those with insurance was lower than that of those without insurance in all ward categories, although this difference is not seen in the median values.

**Table 4:**
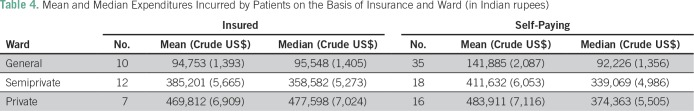
Mean and Median Expenditures Incurred by Patients on the Basis of Insurance and Ward (in Indian rupees)

In the total sample, 76.5% faced a catastrophic impact when the catastrophic threshold was set at 10% of annual household expenditure. Table 5 lists the patients facing a catastrophic impact, segregated on the basis of the ward to which they were admitted.

**Table 5:**
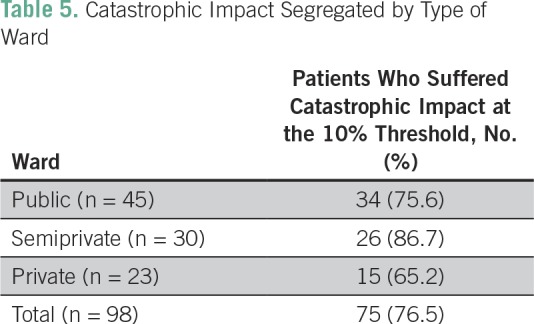
Catastrophic Impact Segregated by Type of Ward

The percentage of patients suffering a catastrophic impact was highest among those in semiprivate wards, at 86.7%, followed by those in public and private wards. If a comparison is made regarding the incidence of financial catastrophe between those with and those without insurance, we see that those without insurance had a significantly higher risk of facing a catastrophic impact compared with those with insurance (*P* < .001). Thirty-eight percent of those with insurance underwent financial catastrophe compared with 93% of those without insurance.

## DISCUSSION

In our study, the total mean expenditure for surgery was found to be Rs.295,679.57 (US$4,348 crude; US$74,420 PPP corrected). This mean amount was significantly higher for patients admitted to private wards and for those with complications. The average amount found in our study is marginally higher than that found in other studies that have analyzed expenditure related to other cancer-related surgeries. Although Mukhopadhyay et al ^21^found the average expenditure for radiotherapy at All India Institute of Medical Sciences to be Rs.36,812, another study, by Nair et al,^22^ which assessed the expenditure by patients receiving cancer treatment in six government hospitals in India, found that although 33.6% of the patients reported receiving treatment free of cost, the average cost of investigations for the remaining patients was Rs.16,739 and for treatment was Rs.41,311. This may be because our hospital is a tertiary cancer center and therefore patients with advanced disease come here for complex treatment, and because PD is a major complex surgery, the cost is often higher than for other cancer surgeries.

The relationship between advanced disease and increased expenditure has been documented by other studies as well. Nair et al^22^ found that expenditure in superspecialty hospitals was higher. Another study in Delhi found that the average expenditure for treatment of oral cancer was Rs.1,49,995 for patients in stage I, Rs.1,41,621 for stage II, and Rs.1,82,859 for stage III. The expenditure incurred for treatment of patients with stage III disease was significantly higher than for those with stage I and stage II disease.^23^ A study by Han et al^24^ in China also found significantly higher treatment costs for patients with stage III and IV compared with those with stage I and II disease. They also found that treatment costs were higher in male patients than in female patients, which is similar to the findings of our study, although not statistically significant. Another interesting finding was that the cost per patient was found to be lower for patients with health insurance than for those without insurance. This is in contrast to the findings of our study, in which the mean expenditure of those with insurance was slightly higher than that of those without insurance, even though no statistical relationship could be established.

An increasing body of research highlights the adverse effects of OOP health expenditure on a patient’s quality of life, compliance, and cancer outcomes.^25,26^ Although our study did not look at long-term patient outcomes and treatment compliance because the time frame for the study was from the first hospital visit to postoperative recovery, it may be assumed that high OOP expenditure may serve as a deterrent to accessing health care, because patients may avoid mandated treatment for fear of incurring costs.^27-30^

It should be noted that our study did not take into consideration the costs associated with chemotherapy and radiation, indirect costs, and opportunity costs. In many cases, these costs may well exceed the cost of the surgery itself. If our study had included the cost associated with radiation and chemotherapy and indirect costs, the mean cost incurred by the patients would be much higher. Approximately 50% to 60% of patients receive adjuvant chemotherapy with gemcitabine and, given the negative results from adjuvant chemoradiation, only selected patients with margin-positive disease received adjuvant chemoradiation..^31,32^

How, then, do our findings compare with other site-specific cancer costs in other low- and middle-income countries? A study conducted in Vietnam that assessed the direct costs associated with breast cancer found the average amount to be US$975 (US$16,688, PPP corrected). The greatest proportion of this expenditure (64.9% of the total) was attributed to chemotherapy.^33^ Patients in our study would also have incurred significant costs before coming to hospital. The study by Mukhopadhyay et al ^21^found that patients spent an average of Rs.14,597 even before going to All India Institute of Medical Sciences All India Institute of Medical Sciences for treatment. Nair et al^22^ found indirect costs incurred for transport, companions, and so forth, to be, on average, Rs.27,248, whereas the opportunity cost (ie, loss of wages because of illness) was Rs.18,165.

India spends approximately 4.6% of its GDP on health (30% financed by the government and 70% financed privately), compared with 14% in Maldives, 29% in Bhutan, 53% in Sri Lanka, 31% in Thailand, and 61% in China. OOP expenditure by people seeking health care still remains the predominant source of private health finance in India, accounting for 71% of private health expenditure.^3,34,35^

Only 29.6% of the respondents in our study had insurance. Most of those admitted to a public ward were covered by government insurance schemes, whereas for those in private wards, voluntary private insurance was found to be the most common form of insurance. Various government health schemes have been launched by both the state and federal governments to subsidize cancer care for citizens, with aid ranging from INR 30,000 (US$441 crude; US$7,551 PPP corrected) to INR 150,000 (US$2,205 crude; US$37,783 PPP corrected). These schemes are for families living in poverty, and access to them requires government-issued proof of income such as the Below Poverty Line card or the Yellow or Orange ration card. A Below Poverty Line card is provided on the basis of census, to identify poor households eligible for social support, whereas ration cards are segregated according to family income: those with an annual family income < INR 15,000 (US$220 crude; US$378 PPP corrected) are given Yellow cards, and those with an annual family income of up to INR 100,000 (US$1,471 crude; US$25,169 PPP corrected) are given Orange ration cards. Families above this income bracket are expected to opt for private health insurance and some may be covered by employer-based health insurance. Only 9% of our study sample had used government-sponsored schemes. Access to government-sponsored schemes has been questioned because eligible beneficiaries have not been able to use the schemes because of a lack of awareness, difficulty in obtaining the necessary documents for enrolment, and insufficient coverage under the scheme.^36-38^ Low insurance coverage in the population may be viewed as a barrier to accessing health care. Hoang et al^33^ also found that an absence of health insurance was one of the main factors limiting uptake. In our study, only 38% of those with insurance underwent financial catastrophe, compared with 93% of those without insurance. So ideally, had a larger proportion of our sample been covered by insurance, the incidence of financial catastrophe would have been much lower. According to different estimates, approximately 40 to 60 million people in India become impoverished annually because of unexpected health expenditure,^39-42^ which could be attributed to the lack of insurance coverage.

The increasing use of technology in investigations and the high cost of imported medical equipment and patented drugs have been pivotal in increasing the cost of cancer treatment. A dearth of adequate screening programs for early diagnosis and a lack of awareness among patients also lead to cancer being diagnosed at later stages, which often leads to an increase in expenditure because of the need for advanced health care measures.

In our study sample, 76.5% faced a catastrophic impact on the household because of expenditure on health care. Although the mean expenditure was highest in the private ward, the catastrophic impact was higher in patients in semiprivate and public wards. The higher income levels of those admitted to private wards may be the reason for this. A study conducted by Engelgau et al^43^ in 2012 found that the odds of suffering catastrophic health expenditure were 168% higher in patients with cancer compared with those with communicable diseases.

The limitations of our study include the low number of study participants and the fact that the study was conducted at a single institute. Because this was a tertiary referral hospital, the findings of this study may not be representative the whole of India.

The costs associated with complex surgery for GI and other hepatobiliary surgeries, although relatively cheaper in India than in high-income countries, are high and often unaffordable for the majority of India’s population. Many people may be denied complex cancer care because of their inability to pay for services. Even though the Indian Government runs many schemes to provide subsidized care to the masses, lack of awareness and lack of necessary documents such as ration cards may cause them to forego health care altogether. With the increasing burden of cancers requiring complex tertiary-level surgical care, new approaches to strengthening the public health care surgical system must be undertaken on an urgent basis in India and in other emerging powers.^4^ New structural models are needed, especially the development of centers of cancer surgical excellence with high volumes to reduce costs and improve the quality of care.
